# The Rhetoric of Healthcare and the Moral Debate About Theatre-Funded Hospitals in Early Modern Spain

**DOI:** 10.1007/s10912-024-09892-8

**Published:** 2024-10-01

**Authors:** Ted L. L. Bergman

**Affiliations:** School of Modern Languages, University of St Andrews, Buchanan Building, Union St, St Andrews, KY16 9PH Scotland, UK

**Keywords:** Public health, Theatre, Hospitals, Morality, Rhetoric

## Abstract

While early modern Spain may seem a world away, it is an extremely rich and relevant context for gaining a better understanding of the Rhetoric of Health, specifically the power of metaphor, in the related spheres of policy-making and public debate. It was a time and place in which the urban populace’s physical well-being depended upon the fortunes of theatrical performances due to a system of alms for hospitals driven by ticket receipts. Anti-theatricalists argued that the immoral nature of theatrical performances made them spiritually and medically detrimental to society. Pro-theatricalists argued that plays were always a public good on balance because they raised much-needed funds for hospitals. Instead of producing a conflict between morality and public health, each side reinforced their connection until the two topics became nearly inseparable in the sphere of public debate. While pro-theatricalists mainly stayed with their arguments about funding hospitals, anti-theatricalists developed a new strategy of literalising the metaphor of theatre as a “plague of the republic” and arguing that immoral entertainment brought literal disease to the populace as a punishment from God. This exemplifies Stephen Pender’s observation of how, in an early modern medical context, “Rhetoric as a way of perceiving probabilities and adjusting one’s argument to the audience and circumstance offers a model of ethical action and interaction”. This article is organised chronologically to track specific adjustments to a specific public-health debate that rely upon moral metaphors of medicine. Each side wrangled over these metaphors in an effort to break a deadlock in a public-health policy debate with entertainment, finance, and morality at its centre. By the end of the seventeenth century, anti-theatricalists finally found their best rhetorical weapon in the literalisation of the “plague of the republic” metaphor, but it only offered a short-term solution to banning theatre contingent upon the ebb and flow of epidemics. Simultaneously, the finance structure of funding hospitals began to erase the role of hospitals from the longstanding debate about the morality of public theatre. The case of early modern Spain provides valuable lessons about the power of metaphor in the Rhetoric of Healthcare that are still applicable today.

In her 2016 study *Treating the Public*, Rachael Ball convincingly argues for how the combined impact of early-modern Spanish theatre in both urban daily life and funding hospitals “expanded the real and discursive spaces in which theatre entertained, influenced, and treated the public” (2016, 154). Ball also signals how in Spain (more than in England) these “discursive spaces” were overrun by public moral debates about funding public healthcare in hospitals through theatrical entertainments deemed immoral by many. These anti-theatricalists often resorted to the tried-and-true rhetorical strategy of invoking a “diseased and overly feminine body politic” (132). While Ball covers some of the basic points of this rhetoric, there is still much more work to be done on the subject. During the last decade or so, the modern connection between healthcare and rhetoric has been heavily studied, especially in relation to public discourse and governmental policy during and after the Covid-19 pandemic. In relative terms, only recently has this interdisciplinary approach been used for analysing early modern examples. Struever (2012, 252) writes that in seventeenth-century Europe, “Most certainly [the perceived gap between theory and practice] is an aporia that rhetoric and medicine share; and, both are beset by accusations of instability, relativism rooted in the ‘impurity’ of their theoretical allegiances”. For this reason, rhetoric is not merely a separate discipline used to enhance the practice of medicine nor only used to promote or decry its effectiveness. Instead, it is intertwined and inseparable as a practice. The “discursive spaces” described by Ball are more than meeting places between the subjects of medicine and rhetoric, or between public health and morality. They create an overlapping *locus* in which separate subjects fuse together and are difficult to untangle. This is especially true in the case of early modern Spain and even more so in the context of the plague years. During those times, the idea of theatre affecting public health could become much more than a mere metaphor for moral decay. Previous studies on the combined subjects of moral debates, theatre, and public health policy in seventeenth-century Spain only make passing reference to the role of rhetoric itself. Conversely, detailed analyses of the role of rhetoric during the same time period in a broader European context seem to have neglected Spain and the Counter-Reformation altogether.^1^

It is a truism that we must understand the past to better interpret the present, and now a new set of tools are available to us in the Medical Humanities. In modern times, Healthcare and Rhetoric have combined to form a relatively recent discipline. In their introduction to *Rhetoric of Healthcare: Essays Toward a New Disciplinary Inquiry*, the editors Heifferon and Brown (2008, 2) write that “Except for groups such as the American Medical Writers Association and health communication and linguistic scholars, few rhetoricians and researchers in technical communication have addressed the languages and texts of health or illness from a rhetorical point of view”.^2^ In the last decade or so, similar calls for this interdisciplinary approach have been made for Canada (Conrad and Cudahy 2010), as well as Spain and all of Europe (Nespereira-García 2015). In a modern context, studying rhetoric and healthcare is mainly for the purpose of informing public policy in the here and now. However, and despite the potential objections of some historians, I believe that present concerns can be used to study the past as well. Pender provides ample justification when he writes that,

Rhetoric as a way of perceiving probabilities and adjusting one’s argument to the audience and circumstance offers a model of ethical action and interaction; rhetorical inquiry informs the practice of private and public virtue, it ensures an effective transfer of remedial efforts between philosophy and medicine … (Pender 2016, 9).

This article will study debates on the morality of theatre in relation to public health with two purposes in mind. First, to effectively fill a gap in the research on early-modern rhetoric and medicine. As Ball (2016, 2) has shown, the dominance of the English Shakespeare in the Western canon has overshadowed the reputation and works of more prolific and (at the time) more widely read Spanish playwrights. Unlike early modern English theatre, the classical Spanish theatre’s reputation does not precede it within today’s Humanities at large, let alone the Medical Humanities. For this reason, the study of *comedias* in a social context has mainly been carried out either in Hispanist literary circles or by Spanish-language historians. Only in recent years do we find welcome contributions by scholars like Ball (2016) and Campbell (2006). For the benefit of the Medical Humanities, and to provide diverse examples of how rhetoric and medicine have worked together throughout history, we cannot exclude Spanish classical literature from the discussion. Diverse examples provide a deeper understanding and lead us to the second and main purpose of this article: to demonstrate how the combined subjects of theatre and morality offer a particularly effective example of how rhetoric “adjusts one’s argument to the audience and circumstance” and “offers a model of ethical action and interaction” per Pender. Spain was theatre-mad during the seventeenth century. In the major cities of Madrid and Seville, the moral debate on theatre became inseparable from matters of public health policy. Beyond that, such inseparability was much more persistent than in England (Ball 2016, 130–131). This provides yet another reason to study a context that has generally been ignored in the Medical Humanities studies outside of Hispanism’s sphere of influence.

Arguments about the morality of theatre and its role in funding healthcare may seem like a distant point of comparison for illuminating present-day examples, but that distant debate actually offers a clear example of a very modern problem, namely how “sentiment, moral values, and language use” interact in public discourse and align with particular political ideologies (Borghouts et al. 2023, 3). The specific rhetorical devices to be studied here can be divided into two related categories mainly based on metaphor: First, the invocation of a figuratively diseased body politic that is made unwell by indulging in immoral entertainments; secondly, the invocation of a literally diseased populace that is made unwell, through divine means by these same entertainments. Pender’s observation of how rhetoric—and for our purposes too, public health policy—“adjusts one’s argument to the audience and circumstance” is apt for Counter-Reformation Spain and for us today. Metaphor is a powerful tool for shaping public perception in matters of public health, morality, and political action combined. In their conclusions after studying the Philippine President Rodrigo Roa Duterte’s militarization of his country’s Covid-19 response, Navera and Bernadas warn that,

On the one hand, frames and metaphors help rally and consolidate support for a public health policy. On the other hand, such strategies realised through frames and metaphors in discourse may reinforce oversimplification, absolutism, and binarism in public health. Second, the media need to constantly interrogate the assumptions behind the use of frames and metaphors. (Navera and Bernadas 2023, 100)

Understanding the power of metaphor is nothing new. Nor is interrogating its use, whether by “the media” or between rhetorical rivals in an open public debate. Seventeenth-century anti-theatricalists understood the power of medical metaphor and how to wield it. More than that, they anticipated Pender’s observation about adapting one’s rhetoric depending on the circumstances while holding fast to medical and public-health imagery. Furthermore, both sides of the debate on the morality of theatre and funding of hospitals knew that they needed “to constantly interrogate the assumptions behind the use of frames and metaphors”, to cite Navera and Bernadas again (2023, 100). Lastly, both sides likely knew that victory was not guaranteed and that the fight to open or close playhouses in major cities like Madrid and Seville would be constant. What made the debate ongoing, the combined unstable foundations of both rhetoric and medicine, is also what made it difficult to resolve. This instability derives from the shared perceived gap in theory and practice for both rhetoric and medicine, as observed by Struever. It allows advocates, whether modern activists or Counter-Reformation moralists, to harness an uncertainty masked by certainty when promoting particular policy decisions. One manifestation of this gap is an atmosphere of “post-truth” that has many precedents, including “the relatively stifling ideological environment of imperial Spain” when it comes to “a rhetoric of warning and a call to action” (Castillo and Egginton 2022, 43). Nowadays, wielding rhetoric in the face of real or imagined medical uncertainty can take extreme forms, as when Philippine President Duterte leveraged his political power to say “shoot them dead” as a response against Covid-19 quarantine violators (Navera and Bernadas 2023, 94). On the other end of the spectrum, the overlapping uncertainties of rhetoric and medicine can be exploited in a more insidious and long-term manner, as with “anti-maskers” who “are skilled in the rhetoric of scientific practices” and who “have proven themselves to be skilled at data analysis and visualization”, (Russell and Patterson 2023, 141). Though distinct in many ways, both of these modern examples involve the literalisation of metaphor, something that was also developed over time by anti-theatricalists in seventeenth-century Spain. During the Covid-19 pandemic, Duterte’s menace of “shoot them dead” was a simple modification of his “war on drugs” metaphor that he had already deployed to fully militarise public policies (Hapal 2021, 226, 230). In a less centralised setting, “anti-maskers” provide a true example of the gap between theory and practice in both rhetoric and medicine by concretizing their perceived social oppression through the literalized metaphor of the mask or something far worse. To cite one such self-taught rhetorician fighting against mask mandates: “I call it a muzzle. There’s a woman who said that the next step would be a leash” (Bornand et al. 2023, 4). Leaving aside the broader philosophical question of “post-truth” *avant la lettre*, this study will concentrate on how the specific literalisation of metaphor of immoral theatre as a “plague of the republic” (Ball 2016, 130) exploits the uncertainty of both medicine and rhetoric in seventeenth-century Spain.

As the end of the sixteenth century approached, people in Spain flocked from the countryside to major cities like Madrid and Seville, seeking employment and a better life. But this also contributed to a higher concentration of sick and dying amongst the urban population. These demographic shifts also led to a high concentration of urban theatregoers. The silver lining to this dire situation was an increased level of ticket receipts that could be directly channelled to hospitals through an arrangement mainly based on religious co-fraternities’ monopolistic control of the playhouses known as *corrales* (Campbell 2006, 36). Campbell and Ball—along with Hispanists Shergold and Varey (1987) and Suárez García (1996)—have neatly documented the financial connections between hospital financing and theatrical production. What has been less studied is the “wary mix of the recognition of theater as an important source of income and concern over its debatable moral qualities” (Campbell 2006, 48–49). There are still more facets to be uncovered of how the reliance on playhouse revenue “was frequently used during this century to defend theatrical activity against the attacks of moralists” (Shergold and Varey 1987, 62).^3^ In more precise terms, Ball explains that, “for anti-theatricalists, the damage to the body politic, made weaker and more feminine with every staging of a play, far outweighed the social good provided to individuals in hospitals and orphanages” (Ball 2016, 139). Campbell’s idea of a “wary mix” merits further study here, because Ball’s term “outweigh” implies a certain variable tension or movement in balance. The result of this tension is a series of adjustments, that is “rhetoric as a way of perceiving probabilities and adjusting one’s argument to the audience and circumstance” to cite Pender again (2016, 9). In this study, the malleability of medical-moral justification, especially through the literalisation of metaphor, will be the main focus. I will take Pender’s arguments from a more local doctor-patience context and place them into one of open debate between anti-theatricalists and pro-theatricalists. The extensive timespan of examples taken for this study, from 1592 until 1720, strongly suggests that neither side ever fully gained the upper hand by way of argument itself. This also suggests that the variability of the rhetoric cannot be linked with any increased effectiveness on either side, but this is a problem that still exists today. Even with modern data available, it is one thing to claim effectiveness for the rhetoric of healthcare on a theoretical level (Naji Aziz 2022), but it is another thing to actually prove it. In fact, the results of a thorough study regarding effectiveness may actually run contrary to initial hypotheses (Blankshain et al. 2023, 168). In this study, no definite claims can be made regarding effectiveness when there were other factors at play that held greater weight in the opening and closing of playhouses. What is clear in terms of rhetoric itself is that each side thought that it had an effective strategy. Each side often believed that modifying old arguments or introducing new ones could improve effectiveness. As the debate passed the half-way point of the seventeenth century, one particular device, that of literalising metaphor, became the anti-threatricalists’ best hope, especially when actual plague made theatre as a “plague of the republic” a reality in the context of Divine retribution.

During the 1600 s in Spain, amongst public debates on the morality of theatre, the most common and practical reason for shutting down *corrales* was royal deaths. In 1598, during a time of economic crisis and spurred by the death of his daughter Catherine Michelle in 1597, King Philip II ordered the closure of *corrales* (playhouses) in Madrid and gathered a group of moralists for further consultation. These esteemed men were happy to issue an opinion (*parecer*) condemning *comedias* in general, leaning heavily on arguments taken from a 1589 treatise written by Jesuit Father Pedro de Ribadeneyra titled *Treatise on Tribulation* (*Tratado de Tribulación*).^4^ Following Ribadeneyra’s lead, the rhetoric of the 1598 *parecer* is almost entirely based on classical antecedents and the sayings of Church Fathers. Medical metaphors had yet to become common currency in the debate. The recommendations in the *parecer* only lightly employ them with references to sins provoked by theatre as wounds and sicknesses, cursorily referring to judgments made by “médicos espirituales” (“spiritual physicians”, Cotarelo y Mori 1904, 396). While there was a lack of awareness concerning the power of medical metaphor, this was not for lack of material to draw upon. Ribadeneyra himself had written 9 years beforehand that distressed people resort to profane entertainments, including theatre,

... because through them they have fun and forget their sorrow, which eats away and consumes their hearts; and they do not see that they are only superficially healed, and that the wound is deep inside. Until it is cut off at the root of sorrow, which is sin, it will always break out and give the fruit of death. (Ribadeneyra 1589, 59v)

In response, the City of Madrid (*Villa de Madrid*) made itself the defender of both recreation and hospitals when preparing its own brief to the King. Unfortunately for them, their answer came too late. The monarch died that year and thus provided the moralists with sufficient cause to close playhouses across the nation in mourning (Suárez García 1996, 59). It is still worth studying the Madrid brief since it establishes a rhetorical baseline that will be built upon and amended throughout the seventeenth century. In practical terms, its rhetoric acknowledges the hospitals’ dependence on the theatre income, but it sees a great risk in replacing what is reliable with the whims of an increasingly less generous public during times of increasing urban problems. In this way, by making plain and contemporary financial arguments related to healthcare, the pro-theatricalist statement also argued that the anti-theatricalists were behind the times in their appraisal of the situation. More importantly, perhaps because of its more proximate association with hospitals, the pro-theatricalists delved deeper into medical metaphors through their rhetoric. They did this by taking advantage of the moralists’ missed opportunity in appropriating Ribadeneyra’s vivid imagery, taking it for themselves, and turning it on its head.

… One ought not to abolish *comedias* just because there have been vices [‘excesos’] in them which can be easily removed. Since one should not take life from a body because it is sick, but rather seek a cure, neither does it seem that, if vice lies in the *comedias*, one should resort to abolishing them but rather remove that vice. (Cotarelo y Mori 1904, 421)

The original surgical metaphor of cutting out sin by removing theatre from society altogether is transformed into a more delicate procedure whereby isolated incidents of vice are surgically removed from theatre without killing the “patient”. Not only is this an example of Pender’s idea of “adjusting one’s argument to the audience and circumstance” (2012, 9), but it also anticipates Navera and Bernadas’s modern exhortation to “interrogate the assumptions behind the use of frames and metaphors” (2022, 100). From the perspective of classical rhetoric, it shows mastery of *antistrephon*, using one’s opponents’ arguments against them, a technique that has proven its usefulness in both modern and early modern times (Lee 2010, 54; Ritchie and Ronald 2001, 61). And if the argument itself is not being appropriated, the same medical knowledge, in this case surgery in an ambivalent context, is certainly being exploited. In policy arguments, either side can exploit what Struever calls “accusations of instability, relativism rooted in the ‘impurity’ of” both medicine and rhetoric’s theoretical allegiances. This *aporia* tied to *antistrephon* is still quite relevant in modern times, as when coronavirus sceptics “create an alternate account of pandemic data visualizations” that “manifested many of the tropes found in traditional scientific discourse” (Russell and Patterson 2023, 139). Back in 1598, policy decisions were supported by the anti-theatricalists initial arguments, but the outright ban was short-lived even as “the 1598 battle set the pattern for many years to come” (McKendrick 1989, 203). Additionally, the wrangling over the use of medical metaphors, what Pender would call “adjusting [of] one’s argument to the audience and circumstance”, was now well underway.

In 1600, the Carmelite Fray José de Jesús María wrote a thorough follow-up response to the original brief from the City of Madrid. We cannot be certain if he had read Ribadeneyra beforehand, but whatever the case, he elaborates upon the bodily metaphor, which had only been superficially referenced in the anti-theatricalist *parecer* of 1598.

… let us concede that theatrical actors are members of this body of the republic, as are its other estates, which is what they are trying to propose here. Then, according to that, one must take notice that when a member of a body is rotten, it is cut off and buried so that it will not corrupt and infect the rest of the body, and the same must be done in this case. (Cotarelo y Mori 1904, 372)

The Carmelite’s adjustment adds the matter of infection and spreading danger, metaphorically referencing real medical observations, based on real case studies like those referenced by Juan Fragoso in his *Cirugía Universal* (Fragoso 1592, 120r). Here, Jesús María is elaborating upon the idea of a diseased “body politic” (Ball 2016, 141–142), a sort of Spanish *corpus reibpublicae misticum* (Swislocki 1999, 49) that can be infected and cured of the moral illness brought on by the *comedia*. The bodily metaphor also lent itself to the idea that God could punish the nation both spiritually and physically.

… The republic does not reap the main benefit that one expects from caring for the poor, which is to please and placate God and keep Him favourable to address all of the republic’s necessities. If anything, with favouring the *comedia*, these alms offend Him and provoke harsh punishments because God does not want plots and inventions of the Devil to accompany works in His name. (Cotarelo y Mori 1904, 378)

This particular argument is deployed here in a mainly religious context but would later be taken up by later anti-theatricalists in a much more literal sense as various plagues hit major population centres. What was once a mostly spiritual admonition would fuse with real-world arguments for protecting public health.

As stated before, it is difficult to determine the exact effectiveness of medical metaphors in the debates on the morality of theatre, but it is clear that adjustments continued to be made, especially by the anti-theatricalists. This occurred even as they seemed to be winning their arguments, at least in the capital of Madrid between 1608 and 1615, a seat of power that could set an example of how to enact policy elsewhere. During this time, the city issued ordinances targeting the immoral mixing of the sexes in the playhouses, a concession to both sides’ agreement that some sort of surgical removal of vice was required. These *ordenanzas* have been extensively cited by scholars of early modern Spanish drama (Shergold and Varey 1971, 13–19) in a bureaucratic context but without much concern for how they fit into a rhetorical landscape. If one considers how they repeat previously failed injunctions, the laws’ actual effectiveness appears quite questionable, meaning that rhetoric is not alone in this regard. Public policy itself, just like the rhetoric and medicine at the time, could suffer from a gap between theory and practice, to cite Struever once again. Nevertheless, both legislators and moralists kept faith in their arguments and continued to fight.

Between the two sets of similar *ordenanzas* of 1608 and 1615, the anti-theatricalists must have felt some encouragement from the actions taken by the civic authorities of Madrid. One opponent optimistically chose to re-litigate points dating back to the original playhouse closures of 1598 while making adjustments in his arguments. This was the Jesuit Father Juan de Mariana, who, in 1609, expanded upon his own thoughts on the evils of theatre originally expressed in his 1599 work *On Kings and the Institution of Kingship* (*De Rege et Regis Institutione*), most famous today for its section on justifiable tyrannicide. Mariana must have thought that there was a particular urgency in joining the public debate because he translated his original work (*De Specaculis*) from Latin into Spanish with his *Treatise Against Public Entertainments* (*Tratado contra los juegos públicos*) (Mariana 1854). Ball has studied Mariana within the larger context of Jesuit paternalism and its claims that theatre weakened the Republic through its promotion of sexual disorder, and she also outlines the Jesuit’s habitual reliance on examples from antiquity (2016, 135–138). In this way, as Cotarelo observed, Mariana’s arguments were out of step and out of time (1904, 430). But even his outdated arguments could not ignore the very contemporary crux of the debate that immoral entertainments were funding an undeniably moral end; sin was being monetised in the name of caring for the sick and dying in hospitals. This explains Mariana; he was forced to make some clumsy adjustments to his antiquated arguments. One strategy was to compare the Roman general Pompeius Magnus’s hypocrisy in disguising a public theatre as a temple of Venus with the hypocrisy of maintaining modern playhouses under the guise of alms for the poor. Mariana also sprinkled his rhetoric with medical metaphors, though mostly by recycling the image of a diseased body politic, that is, “the liberty taken by theatre is a severe plague on Christian behaviour”. When offering his own moral prescription, he feels that it is best to prevent evil appetites from the very beginning, but those who suffer spiritual harm from pursuing illicit pleasure can “at least make sure that the rest are forewarned”, so that they “are at least not infected by this ringworm, this plague” (Mariana 1854, 419). While perhaps not the strongest or most innovative arguments, they demonstrate that even a moralist stuck in the past, and the ancient past at that, had to, in Pender’s words “adjust his argument to the audience and circumstance offers a model of ethical action and interaction”. It was a clear example of how medical metaphors were becoming firmly entrenched in the debate.

As Ball (2016, 133) has observed, fellow Jesuit Father Pedro de Guzmán joined the debate in 1614 with a treatise titled *The Good of Honest Work and the Harm of Idleness, in Eight Discourses* (*Bienes de el honesto trabajo y daños de la ociosidad, en ocho discursos*), the sixth of which focuses on the theatre. Guzmán’s concerns echo those of other moralists, not the least Mariana, and tried his hand at wielding metaphors of public health as a weapon against immoral theatrical performances, especially those by women. Following Mariana, he draws on ancient texts like Tertullian, but he also underscores in his own words the spuriousness of hospital funding as a justification.

What would the ancient Church Fathers say and write on this point, that in order to sustain the poor, cure bodies, one must allow in the Republic an exercise through which many souls fall sick, what they called a school of moral turpitude and an occasion for infinite evils? (Cotarelo y Mori 1904, 341)

The notion of “sick souls” is old, but the context of public welfare and hospitals in an urban setting injects a new urgency into the idea. Repeatedly, sixteenth-century metaphors are renewed in a seventeenth-century battle over the public’s moral and physical welfare, now that theatre is being consumed more heavily than ever before (Meneses 1554, 3v; Ribadeneyra 1592, 229v).

The latest theatre reforms of 1615 did little to quell the attacks on the playhouses as a moral plague. The continued adjustments to medical metaphors, and the newer idea of literalising them, is keenly demonstrated in a manuscript titled *Dialogues on the Comedias* (*Diálogos de las comedias*) dated 1620 by Cotarelo. According to that modern editor, the anonymously written *Dialogues* “differ greatly from other tedious papers that we found ourselves obliged to extract for this bibliographical catalogue” (Cotarelo y Mori 1904, 210). He is referring to two things: First, the high level of detail provided about both religious and profane theatrical productions at the time; secondly, the innovative idea of legislating the inclusion of only honourable people, including priests, as performers, to safeguard the morality of the art form. And yet both Cotarelo and more recent scholars seem to have missed that the 1620 *Dialogues* are also creative in their use of medical metaphor. After mentioning the rhetorical commonplace of theatre as a “plague of the Republic”, the character of the Theologian deploys the word “pestilence” [“pestilencia”] on three occasions. Two of these produce a pair through which a more trite reference to “pestilencia de comedias” is reinvigorated. This is accomplished through a comparison between immoral theatre and legalised sex work described as “this pestilence”. The argument follows thusly that actresses are like sex workers, with both described as “mugercillas” (“silly” or “lost” women) able to attract men and—though it is only implied for actresses—infect those men with venereal disease (Cotarelo y Mori 1904, 213). Thus, the metaphor of infection and disease is nearly brought to a literal plane, something that will eventually happen in the latter half of the 1600s. The author of these *Dialogues* from 1620 also elaborates upon another medical metaphor by establishing an image of the consumption of sinful plays with ingesting poison (“tósigo”). Acknowledging that theatre can be done without promoting sin, he stretches the metaphor further to liken *comedias* to a meal of trout, which eaten fresh “is health and life” but when consumed rotten “is pestilence and death” (213). The Theologian also invokes a metaphorical gangrene limb but adds nothing more. Instead of using it to describe theatre itself, he aims it towards “idle and useless people” who should be “cut off” from the Republic. Much more clever is the *Dialogues*’ use of Saint Augustine’s classical anecdotes from Book IV, Chapter 26 of *De Civitate Dei*, specifically the story of the Roman rustic Titus (“Tito”) also found in other versions by other classical authors. In the early-modern moralist’s revised account, the rustic is visited by a demon who demands that Titus convince the Roman senate to reinstate entertainments that had been suspended on moral grounds. When Titus refuses, the demon “filled him with sickness and humours”. Only after speechifying before the Senate on behalf of these entertainments is he restored to health (Cotarelo y Mori 1904, 219). Here again, the metaphor is nearly literalised, leaving readers with the implication that Divine wrath brings literal illness upon those who consume immoral theatre.

In 1632, the city of Madrid issued a decree (Cotarelo y Mori 1904, 629–630) that repeated the dry administrative wording from a 1616 document dictating the disbursement of theatre profits as alms to hospitals, but without any additional moral strictures. On the administrative front, the anti-theatricalists seem to have gained little ground beyond the *ordenanzas* issued more than a decade beforehand. While some continued to churn out screeds that recycled old theological arguments, there were others who made adjustments in an effort to break the deadlock. One such rhetorical innovator was Fray Jerónimo de la Cruz, who saw the hospital-theatre connection as a particularly entrenched moral problem. In 1638, he published a book titled *The Evangelical, Stoic, and Inspired Job: Ethic, Civil, and Political Doctrine* (*Job evangélico estoico ilustrado: Doctrina ética*, *civil y política*) that sought creative ways to integrate the topic of the day. The work is a heroic effort to combine classical wisdom with biblical doctrine through the figure of a suffering yet stoic job. Within this frame, the mention of “hospitals” would seem surprising if it were not for a problem that had been nagging moralists for over 40 years. Cruz’s analogy goes thusly. God can take away worldly pleasures in the middle of man’s enjoyment, just as He did with Job: “who once a powerful king found himself most miserable beggar in the kingdom, once robust and healthy, became the scabbiest pauper ever found in the hospitals” (Cruz 1638, 48). This seemingly forced anachronism is actually part of a rhetorical long game and anticipates arguments about *comedias* and hospitals that follow 100 pages later. As a way to deal with the frustrating persistence of the debate, Fray Jerónimo creatively channels his exasperation by both indulging his sarcastic side and using the rhetorical device of verbal irony. He is fully aware that pro-theatricalists were continually hanging the good work of the hospitals over the anti-theatricalists’ heads, but to Cruz, the argument reeks so much of false piety that it invokes no less a villain than Judas Iscariot himself.

The second reason made by those who defend *comedias*, that they sustain the hospitals, seems to me like the care that Judas showed for the poor when, for which they would hit him, he wanted that Christ not be given Extreme Unction in life, and that the anointing oil be sold. Begone vice and we will have plenty of hospitals. God does not need the Devil to sustain charity in this world. (Cotarelo y Mori 1904, 156)

Fray Jerónimo is making an oblique and somewhat twisted reference to John Book 12 in the Bible, in which Mary of Bethany anoints Jesus’s feet with oil of spikenard during a dinner at which her brother, the raised Lazarus, is present. A clear point shines through the haze of theological argument though: supporters of theatre are greedy hypocrites whose superficial piety betrays a morally treacherous agenda. To ensure that the readers do not lose the point, they are guided by way of key points listed in the chapter’s heading. The first point is that “That the Consolation of the Inspired Stoic must suffer for God as God presses the Stoic’s hand and He starts to mortify the just” and the last one “That vice causes sickness”. To drive home this last point, the author again employs a double-barrelled Classical and Biblical approach, never losing sight of medical metaphors when citing Seneca’s moral epistle to Lucilius on his health and then Psalms 9:30 and 144:15. The Seneca reference Seneca et al. (2010) includes the question “Quid alios referam innumerabiles morbos, supplicia luxuriae?” [“Need I mention countless other diseases, the punishments of luxury?”] and the combined Psalms contain the lines “Oculi ejus in pauperem respiciunt” [“His eyes look upon the pauper”] and “et tu das escam illorum in tempore opportuno” [“You give them their meat in due time”] (2010, 196).^5^ Lest the meaning of the latter references be lost, Fray Jerónimo spells it out: “so said the holy King, as we can well trust that there will be enough sustenance. Vice begone, there will be fewer vicious people, and much fewer sick people” (Cruz 1638, 156–157). Although they are complicated in their execution, these arguments advance previous methods of combining theology with policy arguments for improving public health by avoiding Divine wrath. With Cruz’s innovations, the metaphor of a diseased body politic is further literalised and begins to approach an effectively mechanical *quid pro quo* whereby. We have reached the point where, according to the moralists, banning immoral theatre will directly impact the physical wellness of the population.

As they continued to elaborate upon their welfare-based arguments against the evils of the public theatre, these moralists anticipated another moment like 1598 when a royal death could give their attacks extra momentum by coinciding with administrative decisions to suspend theatrical productions across the land. As Shergold and Varey explain, the deaths of the Queen in 1644 and Prince Baltasar Carlos in 1646 led to a complete shutdown of performances until 1648. In that year, permission was given for *autos sacramentales*, allegorical plays written for Corpus Christi celebrations, and the renewal of this permission in 1649 and 1650 must have led to the complete return of theatre. Cotarelo’s modern collection of diatribes indicates that 1649 was a particularly rich year for attacking hospital charity as a justification for such a sinful pursuit as attending the theatre. It truly anticipated Pender’s characterisation of “rhetoric as a way of perceiving probabilities and adjusting one’s argument to the audience and circumstance offers a model of ethical action and interaction”. The anti-theatricalists must have seen the theatre shutdown as the opportune moment to strike as they made further rhetorical adjustments to focus more sharply on the hospital-theatre moral conundrum. At this point in history, Pender’s “perceived probabilities” must have seemed to tilt overwhelmingly in the moralists’ favour. Previous strategies became repurposed and intensified. In 1649, Don Luis Crespí de Borja framed his *Response to a Consultation on Whether or not the* Comedias *Performed in Spain are Licit* [*Respuesta de una consulta sobre si son lícitas las comedias que se usan en España*] as a sort of *consulta* like that made after King Philip II’s death in 1598. He also injects the sort of irony that Fray Jerónimo de la Cruz had employed earlier in 1638. Crespí de Borja sarcastically ponders,

What would we not give, for sustaining a hospital for sick Moors in Algiers, to have a place there from which we could preach faith in Jesus Christ? What would heretics [Lutherans] not give so that we would allow them to have a house in which to preach about their sect? Many hospitals could be sustained this way. (Crespí de Borja 1649, 59)

While he does not go as far as Cruz by implying that vices brought by the *comedia* are an immediate cause for sickness, he does repeat the example of Judas cited by his predecessor. Still, amidst the repetition of old arguments, the intent to push the debate further through various rhetorical tweaks remained strong.^6^

If an anti-theatricalist was unable to make major changes in ongoing arguments, he could at least intensify them. In addition to this exaggeration, he could raise his voice further by recasting his entire identity as a sort of “insider” who was especially incensed at the depravity of those in his profession. Likely from the same year that Crespí de Borja published his 1649 “response to a consultation”, we find a “memorandum” (“memorial”) written by someone identifying themself as an actor named Cristóbal Santiago Ortiz. Cotarelo is immensely sceptical of this claim: “The tenor itself of the *Memorial* is basically screaming at us that it is not the work of any actor, because it is a fierce diatribe against them and against Spanish theatre. It belongs, without a shadow of a doubt, to some theologian or moralist…” (Cotarelo y Mori 1904, 541). Ortiz adjusts the standard arguments against funding hospitals through theatre by injecting hyperbole, explaining that,

… the excess of greed and the great necessity of Hospitals and public properties (‘propios’) of the cities has meant building, for the last twenty years in these parts, so many playhouses, that there are few cities, or even towns in the neighbouring areas, that do not have them, and nearly all of them as a source of rents (‘arrendamientos’), which is why there are so many theatre companies with immoral people (‘gente perdida’). (Pellicer 1804, 185). 

This mostly re-hashed argument contains an important if hyperbolic detail: major cities could serve as a template for outlying areas, and Spain in general, when it came to financing hospitals through theatre. Even when taken with a grain of salt, the point must contain some truth. The undercurrent is that major cities were spreading sinful methods of public-health finance, not unlike the metaphorical plague that had been decried in many other harangues. With shrill overstatement as his main contribution to the debate, the supposed actor makes adjustments on another common point: the importance of the spiritual welfare and everlasting life of the population.

And if this reform of the actors does take place, let them close the theatres; since there would less harm in losing the alms for hospitals, and that the public loses its entertainment, so that we not lose so many souls, living in such a bad state, and the majority of them dying in their perverted youth, and so many times in violent deaths. (Pellicer 1804, 187)

Sex and violence, still the bugbears of anti-entertainment moralists in our modern age, are held by this purported actor as a direct threat to the nation when one considers their impact on the afterlife. The shrillness of Ortiz’s arguments, their dependence on heightening what was previously declared, would indicate that the anti-threatricalists were running out of ideas. The pro-theatricalists saw no need to renew their arguments, since the moralists’ lifelong dream of permanently shutting down the theatres, or at least handing complete control of theatre to religious authorities and “honourable men” was not close to being fulfilled. But there was one strategy that still offered hope, and that was the full literalisation of the “plague of the republic” metaphor. There was still this argument to be made: too much theatre could literally kill off the population, and not only in a spiritual sense.

Ortiz’s hyperbolic style relied upon the surrounding circumstances of the 1640s. Aside from the royal deaths cited above, there were wide-ranging catastrophic problems that had to be dealt with like war and plague. In 1644, although the theatres were ultimately shut down under the pretext of the Queen’s death, a decree from the Council of Castile contained amongst its provisions: “…that *comedias* either be eliminated or suspended for now, starting from Palm Sunday until God sees fit to end the wars that Castile finds itself so near…” (Pellicer 1804, 217). James Casey writes that signs of plague started appearing in Andalusia in 1646, but in times of flood, things got much worse, especially in Seville in 1649, “When people were dying at the rate of 500 a day–perhaps thirty times as many as would normally have died in a city that size–the authorities declared a state of plague” (Casey 1999, 38).^7^ With such destruction and despair on people’s minds, it might seem odd that a major source of income for hospitals was being shut down. But it was more than just a matter of stopping frivolity since there were serious matters to attend to. Anti-theatricalists had always argued that theatre was inappropriate or counterproductive distracting during times of crises, but now they were arguing that public entertainments may have actually been *causing* widespread catastrophes that threatened public welfare. A case in point is the satirical poem from 1649, deemed anonymous by Cotarelo y Mori (1904, 544) but attributed to Esteban Manuel de Villegas by Cillero Ulecia (1971, 200). The work is titled *Against the Effort to Once Again Bring Back the* Comedia *to the Playhouses* [*Contra el conato de haber vuelto otra vez las comedias al teatro*]. It contains the following lines evoking a catastrophe of Biblical proportions: “Bellicose War and Pestilence / Each is brandishing its sword: /¡Oh, to have rest in times of tears! / ¿What more could Babylon do, besieged / By Persians and Medes when a lascivious king / profaned the vessels of Holy God?” (Cotarelo y Mori 1904, 545). This is an allusion to the Fifth Book of Daniel and Belshazzar’s Feast, an episode of the Bible in which the king is warned by literal writing on the wall about receiving divine judgment. When addressing the public-health argument, the voice of this poem presents the other side with barely concealed sarcasm. Those in favour of the *comedia* will say: “Also, keep it so those hospitals / are equipped with income to give health / to those who lose it due to various evils. / … / And thus, what greater accomplishment, when the shock / of the fever assaults you, than to find a nest / where you can curl up and recover your strength? (549). The hospital-theatre finance system creates an endless cycle of depravity linked to disease. Pestilence is heavily implied as Divine retribution for the promotion and consumption of immoral theatre. Hospitals that are funded by immoral means provide a cure that is worse than the disease, both physically and spiritually.

In 1649, the archbishop of Seville, Don Fray Pedro de Tapía spoke from first-hand experience when writing about this trap created by the Devil that seemed to make the elimination of *comedias* impossible (Cotarelo y Mori 1904, 564). Unlike the armchair moralists who came before him, he broke through this conundrum by taking pragmatic financial measures when rhetorical ones seem to have failed. According to his biographer, Tapía tirelessly campaigned for hospital funding without the aid of theatres, in effect making a reality out of the easy suggestions made so many times by moralists in the first half of the century. Beyond securing and providing financing for medical care, the archbishop visited hospitals repeatedly, as much as 12 times a week in Cordoba, where he also ordered that the streets be cleaned of plague-contaminated clothing (Lorea 1676, 180). Looking upon the works and efforts of this subject, the biographer Lorea explains that the stakes for the last 50 years remained high. He references the original *parecer* from 1598 and laments, “Oh, the many years that God-fearing men have shouted out against the plague of *comedias* where moral behaviour (‘buenas costumbres’) perish, where chasteness (‘honestidad’) is in danger, a school in which the Devil teaches lascivious profanities and moral turpitude (‘torpeza’)!” (Lorea 1676, 126). Tapía himself, so deeply involved with treating the plague, did much more than his biographer to literalise “the plague of *comedias*”. In a letter dated 1655 and addressed to the “Father Confessor to His Majesty”, the Archbishop bluntly explains that, while there have always been sins, never before have they been committed with such abandon, especially regarding those provoked by watching *comedias*. In a clear allusion to the shutting down of playhouses, he asks that the Most Reverend Father Confessor, “consider that from the year 44 until the year 49 there were no *comedias*, and they were not missed in the Republic, and that affairs during that time were better”. His choice of 1649 as an end-date is significant because this is considered the true plague year in Seville, making the previous 5 years seem good in comparison. Likewise, while the worst of the plague was long over in 1655, Tapía holds onto the idea of a direct connection between the arrival of the plague and the immoral behaviour of his parishioners. He cites a passage from the Book of Hosea, Chapter 4, here translated from the King James Version: “[T]here is no truth, nor mercy, nor knowledge of God in the land. By swearing, and lying, and killing, and stealing, and committing adultery, they break out, and blood toucheth blood”. In a follow-up letter written 3 weeks later, Tapía pushed his arguments further for the Reverend,

since now Kings do not call for (‘no se usa ahora hacer’) public displays of penance (‘mortificaciones públicas’) during public calamities, like David and Jehoshaphat once did, as well as others, including some reprobates like Ahab and the [king of] Nineveh, and they placated God, it is not too much to at least avoid celebrations and *comedias*, even if they were allowed, since no lesser calamities are being suffered right now. (Lorea 1676, 254)

It was clear that the widespread trauma resulting from the epidemic of 1649 had made an impact on the rhetoric of the anti-threatricalists. They were now making their final adjustments, marching towards the full literalisation of the metaphor of theatre as a “plague of the republic”.

Ball (2016, 66) has signalled how plague years and flooding in Seville during the 1640s and 1650s “put added pressure on city hospitals, apothecaries, medical practitioners, public officials, and poor relief efforts”, including how bans on theatre added to this pressure. What was already a disaster for public health and theatre income became a boon for Andalusian anti-theatricalists. Amongst them was the lawyer Don Tomás de Castro y Águila, who, in 1649, published his *Spiritual and Temporal Remedies for Preserving the Republic* [*Remedios espirituales y temporales para preservar la República*]. Like several of his predecessors, he wrapped himself in the imagined garb of a government advisor by appropriating the genre known as the *espejo de príncipes* [“Mirror of Princes”]. In this ostensible guide for the ruling class, Castro eagerly seized upon the plague imagery that must have filled the minds of so many witnesses to the real devastation of disease that year.

And thus the Christian Governor shall first rely upon this remedy to limit cases of mortality and plagues in his Republic, and this advises the aforementioned Father Fray Juan Marqués (d. § 4) and he condemns the opinion of those who these days ask that there be lustful *comedias* and dancing. And he refers us to the fact that Saint Augustine most justly admonished the Romans, who were vainly convinced that by reviving the public games, which they called *Scinicos*, they would limit a certain plague in Rome. He admonished them because the remedy for the plague is not to frequent the theatre, but rather the temples. (Castro y Águila 1649, 24r)

Castro y Águila’s book contains much advice regarding the regulation of plague response and hospitals, and it is significant that his last words on the subject stress the connection between spiritual and physical welfare, the foundational idea behind literalising the “plague of the republic” metaphor. His final thoughts pointedly cite a recently published short treatise by Doctor Pedro Barba, “Physician to the Chamber of his Majesty”, who, along with other experts, “make even more necessary the spiritual and temporal remedies listed in our work, and their execution are of the greatest importance in order to achieve universal health and welfare” (Castro y Águila 1649, 58v).

The mid-century plague years gave the anti-theatricalists serious ammunition for their arguments. By literalising the metaphor of theatre as a “plague of the republic”, with immoral entertainments as a direct cause of disease, they had discovered their own form of “data visualization”, to borrow from Russell and Patterson (2023, 139). But due to the rather limited understanding about the spread of disease, pro-theatricalists were able to step into the same space of uncertainty and wrestle over the same tropes that were more political than scientific. The poet Don Luis de Ulloa y Pereira, a native of the city of Toro and a favoured friend of the extremely powerful Count-Duque Olivares, presents the common argument that *comedias* might need reform, but they never should be banned (Cotarelo y Mori 1904, 575). His strategy was to fight the increasing exaggeration and shrillness of the anti-theatricalists who had gained a second wind in their justifications during the plague years. He reminds readers that audiences are not as gullible and corruptible as opponents might imagine.

Regarding those who attack, and of those who defend the *comedias*, few treat the matter with temperance: but rather each side is equally impassioned, most people use such exaggerations that they themselves discredit their opinions: some say that the *comedia* is a plague of the Republic, a teacher of vices, and the source of all evils. (Ulloa Pereira 1659, 192r)

He significantly appropriates his opponents’ use of sarcasm for his own purposes, citing the exact term “plague of the Republic” in recognition of the metaphor’s recent literalisation and increased rhetorical power, and calling for calmer heads to prevail. Extending his medical metaphor and further promoting the idea of more tempered arguments, Ulloa combines two old ideas into a new one. Recalling the idea that *comedias* can be instructive through negative exemplarity, showing the audience how not to behave, Ulloa compares this to a medicine that can either heal or kill, depending on how it is used: “… teaching a moral lesson (‘escarmentar’) through the bad behaviour of others (‘males ajenos’) it is prudence, keeping them on hand is medicine; while using them poorly is ignorance, or malice” (Ulloa Pereira 1659, 202r). He then deploys sarcasm once again, ironically arguing that “since because one can extract the worst from *comedias,* they must be abolished and so the same must be done with histories, in which both good and bad examples are referenced without them being confused (‘sin comunicación’)” (202v). He is more than merely dismissive of the “plague of the republic” metaphor. Through implication, he is also condemning the anti-theatricalists’ exploitation of a real calamity, the plague, by using heightened rhetoric. Ulloa was fully aware of the moralists’ new strategy and more than ready to take it on.

When King Philip IV died in 1665, playhouses across the country were shut down for a little over a year. The public debate continued to simmer, but like before, it would take an additional calamity, an epidemic, for the medically inflected rhetoric to issue its last gasp of innovation: the full literalisation of the “plague of the republic” metaphor. As in 1598, nationwide mourning and the temporary abolition of theatre were accompanied by a *consulta*, this time in 1666 by the Council of Castile. The majority decision held that *comedias* were—as had been argued by pro-theatricalists many times before—morally “indiferente” and an important source of funding for hospitals. At the same time, theatrical entertainments could effectively be replaced by alternative means of taxation, so the door for their suspension was still left open (Cotarelo y Mori 1904, 176).^8^ In 1672, the Queen Maria Anna of Austria ordered another gathering of advisors (“Junta Superior”) that returned with a series of arguments against public theatre, echoing the dozens that had been published in the decades beforehand. The “junta” was probably wary of the counterargument that abolishing theatre was too extreme a measure, one that ignored the need for hospitals. Surprisingly, they addressed the delicate balance by taking a rather extreme proposition that still managed to safeguard funding. They would not just suspend *comedias*, but also religious *Corpus Christi* plays meant to celebrate the Eucharist. “[B]y taking away the city’s expenditures on the Corpus *autos*, one could give the order so as to supplement the need that hospitals face during the time of this suspension” (Cotarelo y Mori 1904, 388). Nowhere in this document do the officials deploy the “plague of the republic” image, neither figuratively nor metaphorically. Instead, their main arguments rely on straightforward observations about the ineffectiveness of combatting the increased moral laxness brought on by recent entertainments. The extreme measure of complete suspension is one of last resort that now must be taken. Perhaps a more dire warning that incorporated the anti-theatricalists’ latest strategy would have had a greater effect. Cotarelo informs us that “the Queen, nevertheless, did not dare to follow the *Junta’s* recommendation, and *comedias* continued to be performed until 1682, when they suffered a brief suspension due to the plague” (Cotarelo y Mori 1904, 390). One wonders what decision might have been made, and what arguments might have been included in the consultation, if the plague had struck 10 years earlier. The intervening years in Seville give us a glimpse of what could have occurred, but didn’t, at the capital in 1672.

While there was no national ban during the 1670s and 1680s, individual cities could exercise their own discretion. In the case of Seville, particularly hard hit by the plague lasting from 1676 to 1685 (Casey 1999, 37), the cessation of theatre became much more permanent.^9^ According to Cotarelo, 1679 saw the anti-theatricalists triumph, and only in 1767 would theatrical representations return to that Andalusian city. They would then be suspended again in 1775 and only return in 1795 (Cotarelo y Mori 1904, 428). Cotarelo wryly writes that the last act in the life of one of the opponents, Don Miguel Mañara, was to fully intervene in the debate with a letter written in 1679 to a member of the Council of Castile. Benefitting from the horrors of the plague, he had plenty of support for his full literalisation of sickness as Divine punishment meted out for consuming immoral theatre. For him, the connection was clear, and he rhetorically asks, “…seeing ourselves besieged by plague and full of sickness and hunger, had we no other hope than to eliminate these diabolical things from in front of us in order to temper His ire?” His disdain for the Council’s decision to allow *comedias* on a national level is palpable. He rhetorically asks if the Council sides with Christ or with Barabbas, sneering at the pretext of funding hospitals. For Mañara, that old point is only raised by those “interested in this constant deception”. His sarcasm then quickly turns to righteous anger in the letter as he declares,

God is just and jealous of His honour, and if there is nobody on this earth who will reclaim it, and if we lack the officials to do so, they can instead be found in heaven. Just as Saint Gregory of Nazianzus saw one at the castle of Santangelo, sheathing his sword after nearly the entire city had died of the plague. (Cotarelo y Mori 1904, 428)

Having learned from his predecessors, more than augment the shrillness of his rhetoric, he fully embraces the literalisation of the “plague of the republic” metaphor by invoking a medieval public-health “Miracle of Sant’Angelo” that proved the power of piety combined with civic action and leadership (Latham 2015, 24–25).

While we cannot empirically measure the effectiveness of this continued literalisation, its place in the debate became secure and effectively was the last strategy available to anti-theatricalists. Consider the words of a “famous Jesuit missionary” named Father Tirso González de Santalla (Cotarelo y Mori 1904, 327). He, like Mañara, was a tireless campaigner against the evils of the *comedia* for the sake of people’s spiritual and physical welfare. Not only did he contribute, like Mañara, to the banning of theatre from Seville for nearly a century, but his lasting legacy came from curing the city of the plague. A continuation of the seventeenth-century historian Diego Ortiz de Zúñiga’s *Annals of Seville* [*Anales de Sevilla*] by Antonio María Espinosa y Cárcel admiringly recounts the results of González de Santalla’s contribution to the theatre ban in 1672: “Experience shows that the wise judgments of the city had their desired effect, since although the plague continued in nearby towns, God freed this city from contamination” (Cotarelo y Mori 1904, 327). Theatre as a “plague of the republic” was no longer a mere metaphor, but a direct medical cause whose elimination was celebrated as an effective remedy for epidemics. But a previous problem lingered and would never go away, namely what appeared to be effective and what actually was were two different matters.

In the decades of 1680 and 1690, this idea of rhetoric-made-real had been seized upon by both sides of the debate. A 1681 anonymous pro-theatrical argument sent to King Charles II joyously recalls a “harmonious *comedia*” performed in Vienna after the great plague of 1679 had returned. Disarming the literalised “plague of the republic” metaphor, the author ironically argues that “If the *comedia* were the origin of this contagion, then the Catholic heart of the Emperor would have been the quickest in deciding to banish from his dominions this terrible danger” (Cotarelo y Mori 1904, 44). In his 1683 book *Good Zeal* (*El buen zelo*), Pedro Fomperosa y Quintana provides a very contradictory version of the king’s permissiveness and reflects back on how the monarch “whom God shall make prosper with long life and felicitous succession, in order to placate Divine wrath, manifest in a plague, showed his own against Theatres, ordering in his Royal Decree that they be shut down” (Fomperosa y Quintana 1683, 93). But these triumphant words obscure the problem that accompanied the successful literalisation of “the plague of the republic”: the solution that satisfied the anti-theatricalists most powerful and medically based argument was only implemented on a short-term basis. Documents gathered by the historians Shergold and Varey for the years cited above show that there was a theatre shutdown in Madrid from July 1681 until January 1682, but none of these specifically refers to the aim of “placating Divine wrath”. In fact, Fomperosa y Quintana seems to be screaming too loudly about his literalised metaphor because,

On the 8th of January 1682, the King sent an order to the President of the Council in which, as a consequence of the good news coming out of Andalusia about how the plague had ceased, he ordered that theatre once again be performed so that the people have some relief (‘desahogo’) and that the hospitals not lack from the convenience that follows from what is done in the playhouses. (Shergold and Varey 1974, 14)

Recognising the literal medical danger of the “plague of the republic” was now commonplace and could perhaps affect policy but not in any lasting sense. The pro-theatricalist Tomás de Guzmán was quick to strike back at Fomperosa y Quintana’s hyperbolic and impractical zeal. In the same year as his opponent’s diatribe, he asked readers to “take a look at *Good Zeal* since the title of ‘zeal’ does not apply, nor ‘good’, but rather a Mewling Zeal, that irritates with its tuneless, prolix and repeated screams arising from his blind, irrational and jealous passion” (Guzmán 1683, 11).

As the seventeenth century drew to a close, the theatre-plague connection as a response to the theatre-hospital justification remained strong in a rhetorical sense and lined up nicely with the occasions of actual plague. But the short-term closures of theatres betrayed that financial considerations were an overriding consideration, even when the rhetoric seemed to be working. In 1694, the tireless campaigner against immoral theatre in the city of Cordoba, Father Francisco de Posadas, finally got his wish. Using the pretext of denying the entry of a theatre troupe by the impresario Francisco de León after a suspension of *comedias* due to military setbacks in Cataluña and excessive heat during the summer, Father Posadas argued for a true permanent ban. This was approved by the city council (García Gómez 1990, 38n) and is framed by his eighteenth-century biographer as proof of his subject’s rhetorical master. The Father’s sermonising advocacy included the following argument for the city’s leader:

Sir, if the plague were to arrive at this Republic and ask for entry, promising that it will not infect anybody (‘no contagiar a nadie’), and offering alms to some Hospitals in exchange for entry, it should not be admitted under that condition. With even more reason should *Comedias* be expelled, even if they do not infect, because they are, like Saint Isidore says, a plague of the Republic and can bring people to ruin (‘apestar’). (Alcalá 1728, 214)

The implication here is that the literalisation of the “plague of the republic” metaphor was a triumph, but the exact impact of the rhetorical campaign is severely questioned by the modern historian Ángel María García Gómez. He sees financial factors as a much more important motivator for the city council and “one has to ask why the sparse opposition raised by the theatre shutdown finds an immediate response in very concrete plans to convert the theatre into market building” (García Gómez 1990, 29).

The anti-theatricalists’ mastery of the medical metaphor was undeniable, but so too was an increasing series of overriding non-rhetorical factors at the start of the eighteenth century. The War of the Spanish Succession, which lasted from 1701 until 1714, threw the business of theatre across Spain into disarray. It is significant that Cotarelo’s collection contains only three examples of debates on the morality of theatre during this time period. The only relevant example is in a 1708 letter to King Philip V from Cardinal Don Luis Belluga y Moncada. There, he sums up an entire century of arguments against theatre, evoking past moralists, accumulating citations from Church Fathers, and ending a section of his diatribe with the clipped epithets of “seat of pestilence” from Saint Clement of Alexandria and “plague of the universe” from Saint Isidore of Pelusium (López 1814, 349). Then, without fully literalising the metaphor, he argues that God will be deaf to our prayers in future calamities, if theatre is left to continue in his city of Murcia. Perhaps because he was occupied with the more serious matter of the War, the king waited until 1715 to ask for further religious arguments on the matter. Belluga answered using purely spiritual arguments without the customary medical metaphor to call for a ban on *comedias* and dances, perhaps because there was no real danger of plague at the time that could be used to further support his views. Dances were banned by royal decree in 1718, but the matter of *comedias* was put on hold (López 1814, 355). Finally, a real plague came along, and once again, the literalisation of the “plague of the republic” argument took effect. No less than a royal decree from 1720 stated the following:

Due to the plague at Marseille that is spreading and setting its fire to other places in France, and given that it is not just that, when God’s wrath threatens with such anger, one think of diversions and celebrations, but rather do penance to soothe the scourge with which He threatens us, the King has resolved that for now, in the capital and the entire kingdom, there cease any *comedia* performances or bullfights. (Cotarelo y Mori 1904, 639)

The Great Plague of Marseille in 1720 was the last large-scale epidemic in early modern Europe (Wilson Bowers 2013, 99), and as the scare of such calamity faded entirely, so did the hopes of moralists for a permanent nationwide theatre ban. A royal decree from the following year declares that the entertainments can return, provided that “in them one does not say nor do any unsavoury indecent things that cause a scandal or bad example” (Cotarelo y Mori 1904, 639). Banning theatre had become a stop-gap remedy, not a long-term strategy for maintaining public health through divine means. Attempts were still made at a local level but with sporadic success, and never with permanent effect. Cotarelo y Mori recounts the following for 1721 in Pamplona:

… the aforementioned city saw itself threatened by the scourge of the plague, and as a way to beseech Heaven to liberate from this scourge, the city and the vicar-general agreed, without the consent of the citizens, to make a perpetual vow to never put on nor allow *comedias*, nor allow any acting troupes (‘compañías de farsantes o histriones’). (Cotarelo y Mori 1904, 411).

To answer the citizens’ uproar over this, the Pope himself intervened to nullify the vow (“voto”), which was then appealed by the anti-theatricalists. The pro-theatricalists then responded by adding that the Catholic King himself (Philip V) had said that allowed for honest theatre to be performed, which this certainly was, they argued (639). The Pope sided with the pro-*comedia* camp, provided that they paid 500 *escudos* in alms, and eventually this side one. This case serves as a good example of how, even in the rarefied air of plague-prevent and arguments with the Pope, financial factors across Spain would have a greater effect on the debate than either side having the upper hand in a rhetorical-moral sense. In the 1730s, the theatre-hospital connection began to fade away where it had started, in the capital of Madrid. Varey and Davis explain that this was due to two related reasons: the deterioration and eventual demolition of the city’s original patio-based playhouses, to be replaced by purpose-built *coliseos* in 1736 and 1745, and changing tastes in theatre that made these *coliseos* desirable. Importantly, the city took on the administration of the theatres and paid the hospitals out of a general fund that included profits from the playhouses, a fact to which the hospitals became resigned (Varey and Davis 1997, 51). Thus, pious works could still benefit from immoral entertainments, but in a much less direct and much less condemnable way. Moralists returned to the more standard spiritual and ethical injunctions that had existed for centuries before any co-fraternities decided to fund public health through theatre, co-fraternities that were now effectively out of the theatre business. As a consequence, medical metaphor, especially in literalised form, was no longer a weapon of choice amongst the anti-theatricalists in the ongoing debate on the morality of theatre.

In their article “Rethinking Figurative Language in the Rhetoric of Healthcare Allocation”, focusing on how figurative language can erase a patient’s personhood, Brian Li and Marta Napiorkowska warn that,

Figurative language is also reductionist, normative, and lacking in nuance, distorting the realities of a moral problem. It is often based on hypothetical situations rather than those normally encountered in actual hospital settings. If figurative language is grounded in a concrete source field, it would be tempting to think that these reductions are also absolute truths. (Li and Napiorkowska 2020, 2)

The case of Spanish anti-theatricalists demonstrates how the combined spiritual and physical danger of immoral theatre became increasingly “grounded in a concrete source field” through the threat of real plague. From a modern perspective, the “temptation” to literalise is morally suspect, but from the early-modern perspective, it was morally motivating. By literalising the “plague of the republic” metaphor over time, anti-theatricalists believed that they were purely ethically driven. To protect the spiritual and physical welfare of the population, they saw their arguments as the embodiment of Pender’s modern characterisation of “rhetoric as a way of perceiving probabilities and adjusting one’s argument to the audience and circumstance offers a model of ethical action and interaction” in a public health setting.

## Data Availability

The articles does not contain any datasets or data kept in a particular repository. All data is available in published sources, including early-modern books, which may be available in scanned form online, as in Google Books.
